# Harmine suppresses bladder tumor growth by suppressing vascular endothelial growth factor receptor 2-mediated angiogenesis

**DOI:** 10.1042/BSR20190155

**Published:** 2019-05-02

**Authors:** Cai Hai-rong, Huang Xiang, Zhang Xiao-rong

**Affiliations:** Department of Urology, Ruian People’s Hospital, Wenzhou, Zhejiang 325200, China

**Keywords:** angiogenesis, bladder cancer, harmine, tumor growth, VEGFR2

## Abstract

Angiogenesis is a vital step during the process of oncogenesis of a lot of tumors, with no exception in bladder cancer. One of the useful strategies for the development of new drugs against cancer is targeting angiogenesis. In the present study, we found that a small-molecule natural product, which belonged to the β-carboline alkaloid, named harmine, could strongly inhibit tumor angiogenesis thus exhibiting its ideal treatment efficacy in bladder cancer. *In vivo* study verified that harmine had the effect of inhibition on human bladder tumor xenograft growth. The inhibitory effect of harmine to bladder cancer growth was coordinated by the effects shown on angiogenesis. To further explore the pharmacological activities of harmine, we tested harmine’s influence on blood vessel formation and found that harmine effectively blocked the microvessel sprouting in rat aortic ring assay when stimulated by vascular endothelial growth factor (VEGF). Furthermore, harmine inhibited human umbilical vein endothelial cell (HUVEC) proliferation as well as chemotactic motility, and when we treated HUVEC cell with harmine, the formation of capillary-like structures was also restrained. Moreover, harmine induced bladder cancer cell apoptosis through triggering the caspase-dependent apoptotic pathway and the downstream vascular endothelial growth factor receptor 2 (VEGFR2) kinase pathway was down-regulated, thus suppressing tumor development signals. Herein, our study demonstrated that natural product harmine might have potential in curing human bladder tumor because of its pharmacological function on tumor angiogenesis, trigged by VEGFR2 signaling pathways.

## Introduction

The most common malignant tumor in the urinary system is bladder cancer which has a significant morbidity and mortality [1]. In today’s world, almost more than 380000 new cases are being diagnosed as bladder cancer every year, which amounts to the first incidence of whole urinary diseases [2]. In China, bladder cancer accounts for the eighth rank of all cancer-related mortalities. Although there is a relative lower incidence in China than in the western society, it is still in the face of great difficulties to cure bladder cancer. Benefiting from radical cystectomy and chemotherapy, approximately 35% of the patients suffering from local bladder cancer can survive above 5 years. Unfortunately, the survival rate for those patients with distant metastatic bladder cancer is only about 6% [3]. The curative results still take a dim view compared with other kinds of tumors such as breast cancer or prostate cancer. In a word, it is extremely urgent to improve the therapeutic effect of bladder tumor.

During the process of angiogenesis, there is a physiological course that some old lumping vessels reach out some new minor vessels and then new vessels are formed. It is especially important for the blood and nutrition supply when a tumor develops, thus it functions as a very important step in tumor genesis such as tumor growth, cancer cell invasion, and tumor metastasis [4–8]. Many cell growth factors and cytokines are involved in the process of angiogenesis, among which the most essential one is named vascular endothelial growth factor (VEGF) [9]. It is a member of growth factor/VEGF family and encodes a disulfide-linked homodimer protein product, which always serves as a glycosylated mitogen, and especially has mitosis-promoting effects on endothelial cells [10]. In most cancers, VEGF acts as a positive stimuli to trigger angiogenesis, which is an essential process for tumor development [5]. VEGF signaling pathway is initiated by the first step when it recognizes and binds to its receptor, a tyrosine kinase named vascular endothelial growth factor receptor (VEGFR). However, the VEGFR involved in angiogenesis is usually mediated by VEGFR2 [11]. In detail, the activation of VEGFR2 stimulates many different intracellular signaling molecules. Among them, the most common downstream signals include Src family kinase/focal adhesion kinase [12], phosphoinositide 3-kinase/AKT kinase, mTOR/ribosomal protein S6 kinase [13], protein kinase C/protein kinase D [14], and mitogen extracellular kinase/extracellular signal-related kinase [15], and so on. These signals have vital relationship with the growth, migration, differentiation, and survival of endothelial cells. Because of the potential of curing cancer by blocking VEGF signaling pathway, an antibody against the VEGF – named Avastin – has been approved for the treatment of cancer and up till now has achieved some curative effect in many kinds of cancers [9]. That means some safe and efficacious small molecules have activity against VEGF pathway in suppressing tumor angiogenesis, which will have great application prospects in treating tumor.

Harmine, one of the β-carboline alkaloids that are present in many plants, is extracted from Pergamum harmala seeds. Some previous reports have proved that harmine exerts the activity of inhibiting many intracellular kinases, such as dual-specificity tyrosine-regulated kinase-1a (DYRK1A) and monoamine oxidase-A (MAO-A), and it also inhibits CDC-like kinases [16]. Other reports reveal that harmine has the function of both antitumor and antidepressive as well as protects against type 1 and type 2 diabetes through many different and broad spectrum mechanisms [17–19]. Especially harmine and its derivates could block many kinds of carcinoma cells proliferations such as gastric adenocarcinoma, lung cancer, and leukemia [20]. However, up till now, the exact effect of harmine on bladder cancer and its intrinsic mechanisms have not been adequately revealed. On behalf of those reported research, we hypothesized that harmine might have some significant effects in treating bladder cancer. Thus, we tested the potential anticancer activity of harmine in bladder tumor growth and angiogenesis, and its function and mechanism were elucidated in human bladder cancer cells.

## Materials and methods

### Reagents and antibodies

Harmine (Sigma-Aldrich, St Louis, MO, U.S.A., with purity ≥ 98%) was purchased and its purity was verified by HPLC. Some other reagents used for cell culture such as fetal bovine serum (FBS), trypsin and medium were all purchased from Gibco Life Technology (U.S.A.). Antibodies specific for β-actin, VEGFR2, phospho-VEGFR2 (p-VEGFR2), AKT, ERK1, PARP, cleaved PAPP, and antibodies against Caspase 3 and β-actin were obtained from Cell Signaling Technology. MTS Detection Kit was purchased from BD Biosciences. VEGFR2 signaling pathway agonist VEGF was purchased from Sigma-Aldrich.

### Cell culture

Some typical human bladder carcinoma cell lines are used for *in vitro* experiments, such as RT112, RT4, SW780, BIU87, and 5637. We employed an immortalized normal human urothelial cell SV-HUC-1 as normal control to test the biosecurity of harmine. The cells were purchased from the American Type Culture Collection (ATCC, U.S.A.). All cell lines were cultured in DMEM high glucose or F12K medium containing 10% FBS. Cells were cultured in an incubator filled with 5% CO_2_ at 37 °C. And we used another human normal cell, the primary human umbilical vein endothelial cells (HUVECs) (Science Cell Research Laboratories, San Diego, CA) as a cell model to mimic the process of angiogenesis. HUVECs were cultured in Endothelial Cell Medium (Gibco Life Technology, U.S.A.) supplemented with 5% FBS, 1% endothelial cell growth supplement (ECGS), and 1% penicillin–streptomycin and placed in incubator filled with 5% CO_2_. Every 2–3 days, the medium for cell culture was refreshed.

### Human bladder cancer xenograft

Referring to a previous study [21], we performed the xenograft mouse model assay of human bladder cancer-used RT4 cell line. 5-week-old male BALB/c nude mice with the body weight of about 25 g each were employed and all these mice were randomly divided into two groups. RT4 cell is a typical bladder cancer cell with great tumorigenicity and can be used as an ideal cell model for *in vivo* experiments, thus RT4 cells were injected subcutaneously into each mouse (with RT4 cell number about 2 × 10^6^ per mouse). When the average volume of each tumor grew to 100 mm^3^, mice were administrated with or without harmine (10 mg/kg/day) for a month by intraperitoneal injection daily. After 30 days, all the two groups of mice were killed and dissected, and the solid subcutaneous tumors were stripped, taken photos, and tumor weight or volume were calculated.

### Histology and immunohistochemistry

The tissue sections (5 μm) underwent antigen retrieval by microwave after deparaffinization and rehydration for 10 min in sodium citrate buffer. Then the sections were treated with 3% H_2_O_2_ for 10 min and blocked with 5% goat serum for 1 h at room temperature and then were incubated at 4°C overnight with primary antibodies as follows: 1:100 p-VEGFR2 raised in rabbit. Then the sections were washed in PBS and incubated with the secondary antibody for 30 min. The sections were incubated with 3,3-diaminobenzidine (DAB) as substrate for 3 min. For evaluation, photomicrographs were taken with a digital camera. The stained cells were analyzed by Image-Pro Plus software.

### MTS assay

Cells (5000/well) were seeded in 96-well plates for 72 h. Then cells were treated with 10 µM harmine. According to the manufacturer’s instructions, we assessed the cell viability with an MTS assay kit (Promega, Madison, WI). The absorbance value of the live cells residing in the 96-well plates was measured at 515 nm on a microplate reader (Thermo Fisher).

### *In vitro* migration and invasion assay

To determine the suppression of harmine to HUVECs, the Boyden chamber assay and wound-healing assay were carried out with modifications previously described [22]. HUVECs were starved in advance (4 × 10^4^/well) in 100 µl ECM deprived of FBS and harmine were pipetted into upper chambers (8 µm, BD Biosciences) coated with 0.1% gelatin at different concentrations, while the bottom wells with 600 μl ECM contained 0.5% FBS, 50 ng/ml VEGF, and harmine at accordant concentrations with the upper chamber. When cells were treated for 4–6 h and some cells have migrated from the upper chamber to the bottom, experiment was ceased and cells were fixed with 4% paraformaldehyde for more than 30 min, using a cotton swab, non-migrated cells that were still located in the upper chamber were removed gently, and cells migrated to the bottom were stained with 1% crystal violet. The images were photographed by OLYMPUS inverted microscope. Cells that successively migrated from the upper to the bottom could be counted by randomly selecting five different horizons.

### Rat aortic ring assay

According to a previous study [23], the rat aortic ring assay was performed. Aortas separated from the rat thoracic aorta were cut into rings with a diameter of 1-1.5 mm and were seeded in Matrigel-coated wells randomly. Thin rings were covered with another 100 µl of Matrigel and allowed to solidify for 30 min, then MCDB131 without serum was added. 24 h later, the upper medium was replaced with MCDB131 contain 2.5% FBS with or without different concentrations of harmine. The medium was refreshed every other day. After 5 days, microvessel sprouts were captured via OLYMPUS inverted microscope.

### *In vitro* tube formation assay

Fifty microliters of Matrigel (BD Biosciences, Franklin Lakes, NJ) per well (Corning) were pipetted into precooled 96-well plates and polymerized at 37°C for 30 min. HUVECs (6.5 × 10^3^/well) were seeded into Matrigel-coated plates and treated with various concentrations of harmine containing 50 ng/ml VEGF after starvation for 6 h; culture medium without VEGF was treated as negative control. After 10 h, tubulogenesis was photographed and numbers of tubular structures were counted manually. Each experiment was performed independently in triplicate.

### Apoptosis assay

Using the Apoptosis Detection Kit (BD Biosciences), the apoptosis assay was assessed to justify the effect of harmine to bladder cancer cells. After treated with harmine for 48 h, RT4 cells were collected, washed, and stained with annexin V-fluorescein isothiocyanate and propidium iodide (PI), and were analyzed by flow cytometry (FACS Calibur; BD Biosciences).

### Western blot analysis

RT4s or HUVECs were pretreated with or without various concentrations of harmine for 1–2 h, then HUVECs were stimulated with VEGF_165_ (100 ng/ml) for 5–20 min. Total protein extracts were obtained by RIPA lysis buffer (50 mmol/l Tris–HCl, 150 mmol/l NaCl, 5 mmol/l ethylenediaminetetraacetic acid, 1% Triton X-100, 1% Sodium deoxycholic acid, 0.1% sodium dodecylsulfate, 2 mmol/l phenylmethylsulfonyl fluoride, 30 mmol/l Na_2_HPO_4_, 50 mmol/l NaF, and 1 mmol/l Na_3_VO_4_) for 30 min on ice. Equal protein of each sample was subjected to SDS–PAGE (6–12%), transferred to nitrocellulose filter membranes. Blots were incubated with specific antibodies following the blockage for 1 h at room temperature with 5% FBS. Finally, blots were examined by LI-COR Infrared Imaged Odyssey (Gene Company Limited).

### Ethics statement

The study protocol was approved by the Institutional Review Board of Ruian People’s Hospital. The animal studies were performed after receiving approval of the Institutional Animal Care and Use Committee of Zhejiang University (IACUC approval number 2016-002). All efforts were made to minimize suffering.

### Statistical analysis

All statistical results were analyzed according to the Student’s *t* test except for the statistical analysis of tumor weight which was conducted with two-way ANOVA. Each experiment was performed triplicated. A value of ^*^P<0.05 represented as statistically significant.

## Results

### Harmine inhibits bladder cancer growth and blocks VEGFR2 pathway in the xenograft model

**Figure 1:**
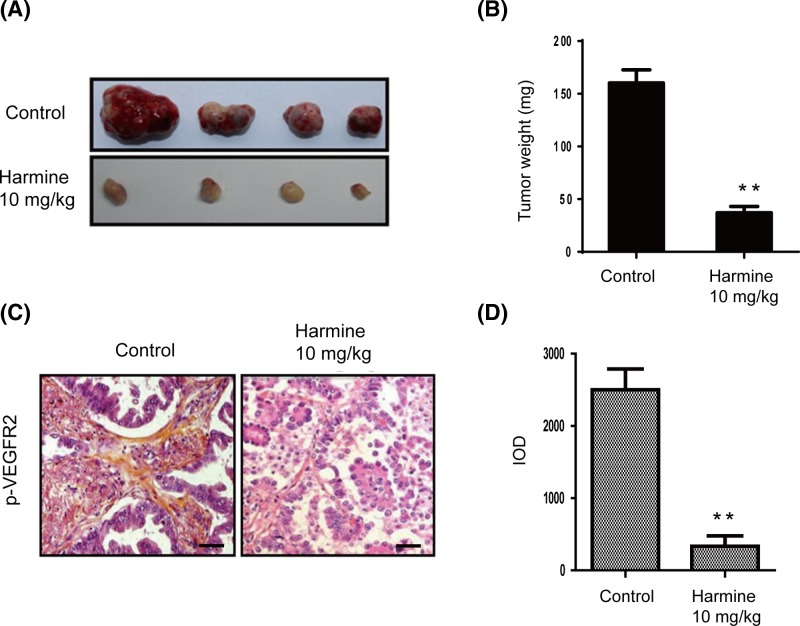
Harmine inhibits bladder tumor growth and VEGFR2 signaling pathway *in vivo* (**A**) The photos of tumors in harmine treatment group and control group. (**B**) Statistical analysis of Figure 1A. (**C**) Immunochemistry staining revealed that harmine inhibited VEGFR2 signaling pathway by blotting p-VEGFR2. Tumor sections were stained using p-VEGFR2 antibody. Bars = 50 um. (**D**) Statistical analysis of Figure 1C. ^**^P<0.01 versus control.

The xenograft bladder tumor model was used to explore the influence of harmine on tumor growth or angiogenesis. When treated with or without harmine, mice received the same dose of harmine (10 mg/kg/day per mouse) or the same volume of DMSO, the solid tumor and their volume and weight were observed. Compared with controls, xenografts grew more slow and small in the harmine-treated group (Figure 1A). The tumor weight in the control group increased to about 150 mg after 30 days; whereas, in the harmine-treated mice, it only reached to about 45 mg (Figure 1B). This result indicated that the proliferation of tumor was greatly inhibited by harmine administration. We also wondered about the mechanism of harmine-inhibited tumor growth, thus we used a VEGFR2 antibody to stain solid tumor sections to test if this inhibition has some relationship with tumor angiogenesis. As shown by the IHC results, the average number of positive p-VEGFR2 in harmine-treated group was significantly decreased compared with untreated group (Figure 1C,D), suggesting that harmine might inhibit tumor growth partially due to its inhibition on tumor angiogenesis.

### Harmine inhibits the proliferation and migration of bladder cancers

**Figure 2:**
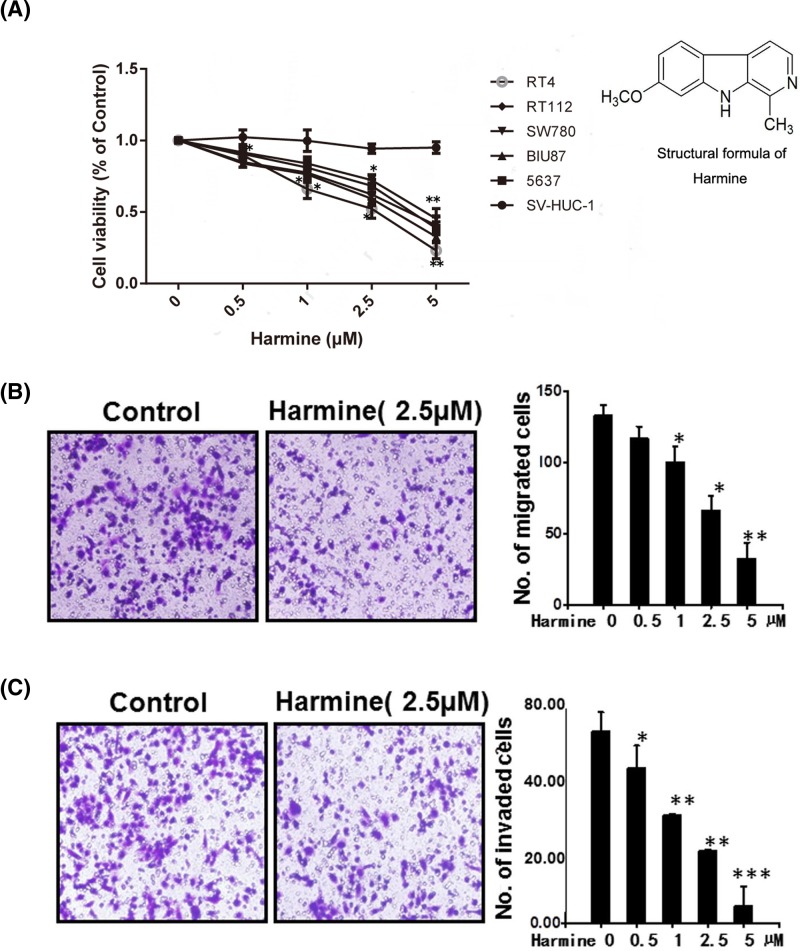
Harmine inhibits bladder cancer cell proliferation and blocks HUVECs migration and invasion (**A**) Harmine inhibited different types of bladder cancer cells proliferation. The cell viability was determined by MTS assay according to Materials and methods. Data are shown as means ± standard deviation from triplicate experiments. ^*^P<0.05 versus control. (**B**) Harmine suppressed HUVEC migration. HUVECs were seeded in transwell chambers and then deprived of FBS for 6 h. After that, cells were scratched by pipette and treated with or without 10 ng/ml VEGF and indicated concentrations of harmine. (**C**) Harmine impaired HUVEC invasion. After HUVECs seeded in the upper chamber of a transwell, the cells were deprived of FBS and then treated with different concentrations of harmine. Data are shown as means ± standard deviation from triplicate experiments. ^*^P<0.05, ^**^P<0.01 versus control.

Figure 2A shows the structural formula of harmine. In the present study, we first tested the influence of 10 µM harmine for 72 h on bladder cancer cells RT112, RT4, SW780, BIU87, 5637, and SV-HUC-1 (normal urothelial cells) by MTS assay. Treating bladder cancer cells with harmine distinctly suppressed the viability of bladder cancer cells, but the effect of harmine on normal bladder cells is somewhat inconspicuous (Figure 2A). The IC_50_ value for harmine inhibition of bladder carcinoma cells’ cellular proliferation was approximately 5–10 μM, while that of SV-HUC-1 cells was much more than 10 μM, indicating that harmine was more toxic on cancer cells than on normal urothelial cells. In summary, harmine inhibits the viability of bladder cancers.

The migration of endothelial cell is a major step of angiogenesis. Therefore, migration assay (Figure 2B) and invasion assay (Figure 2C) were used to test whether harmine affect the motility of endothelial cells. The results indicated that harmine dose-dependently inhibited endothelial cell motility in migration assay and invasion assay.

### Harmine inhibits microvessel sprouting and vascular tubulogenesis of endothelial cells

**Figure 3:**
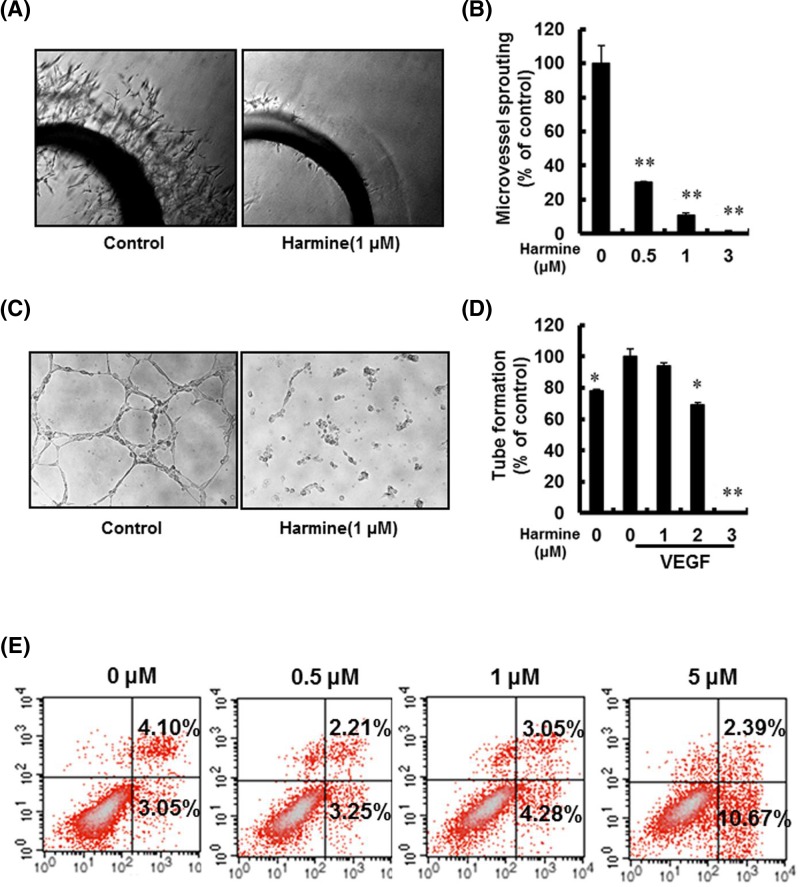
Harmine inhibited the VEGF-induced microvessel sprouting and tube formation of HUVECs and induced RT4 bladder cancer cells apoptosis (**A**) The vessel rings were treated with 20 ng/ml VEGF in the presence or absence of harmine for 6 days. Representative photographs of endothelial cell sprouts forming branching cords from the margins of aortic rings. (**B**) Sprouts were scored from 0 (least positive) to 5 (most positive) in a double-blinded manner. Bars, SD. ^*^P<0.05; ^**^P < 0.01 versus VEGF alone. (**C**) Harmine inhibited the VEGF-induced tube formation of HUVEC tubes. HUVECs were placed in 24-well plates coated with Matrigel (4 × 10^4^ per well). After 6–8 h, cells were fixed and tubular structures were photographed. Magnification, 100. (**D**) The statistical results of Figure 3C. Columns, mean from three different experiments with duplicates; ^*^P<0.05; ^**^P<0.01 versus VEGF alone. (**E**) After incubation with different doses of harmine for 48 h, RT4 cells were collected and apoptosis was assessed by annexin V/PI staining and flow cytometry.

Rat aortic ring assay is a classical assay to study all particular steps in angiogenesis such as endothelial cell activation, pericyte acquisition, migration, and remodeling. To investigate the anti-angiogenic effect of harmine, the aortic ring assay was used. A large number of microvessels sprouted from aortic rings on the fifth day after the rings were embed in Matrigel (Figure 3A) in control group. In contrast, harmine significantly reduced the sprouting in a dose-dependent manner (Figure 3B). Endothelial cells spontaneously formed 3D capillaries exposed to matrigel, this characteristic can be applied to mimic human angiogenesis. Using the 3D vascular tube formation assay, we examined the effect of harmine on tube-like structure formation of endothelial cells, HUVECs treated with different concentrations of harmine were added onto the surface of Matrigel. Our research showed that the vascular tubulogenesis were completely inhibited by harmine at the concentration of 3 μM (Figure 3C,D). Taken together, these data suggested that harmine suppressed angiogenesis.

### Harmine induces bladder cancer cell apoptosis

For the mechanism study of harmine’s inhibition on bladder cancer cell, flow cytometry analysis was performed on RT4 cells. There was a significant increase in the number of apoptotic cells. And necrotic cells were also amounted, both of which were consistent with the increase of harmine concentration, after harmine treatment for 48 h (Figure 3E). With harmine’s concentration increasing from 0 to 5 μM, the percentage of dead cells raised from 7 to about 13%. Anyhow, our data demonstrated that harmine had an inhibitory role on the growth of bladder cancer cell lines.

### Harmine triggers caspase-dependent apoptotic pathway and inhibits VEGFR2-mediated signaling pathway

**Figure 4:**
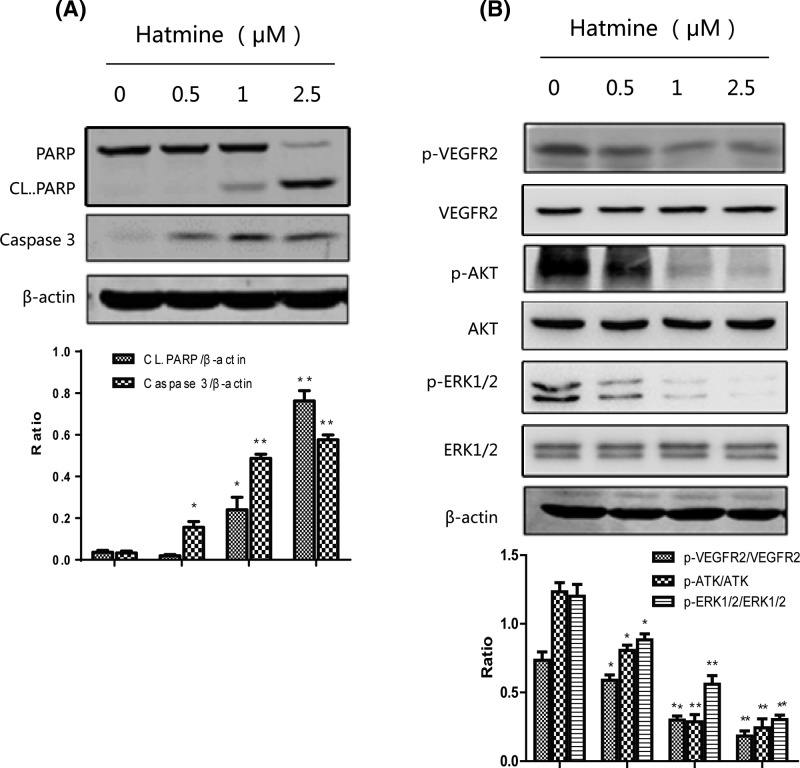
Harmine suppresses bladder cancer growth through inhibiting the VEGFR2 signaling pathway (**A**) Harmine induced cell apoptosis. Proteins from RT4s treated with indicated concentrations of harmine for 24 h were submitted to Western blot for the immunoblotting of caspase-3 and cleaved PARP. (**B**) Harmine inhibits VEGFR2 activation and down-regulated its downstream signals. Harmine suppressed the activation of VEGFR2 in HUVECs. The activation of VEGFR2 from different treatments was analyzed by Western blotting and probed with anti-p-VEGFR2 antibody. The expression of AKT, ERK were also decreased by the treatment of harmine. Western blotting was conducted in a way described in Materials and methods and specific antibodies were used accordingly.

On molecular level, we detected why harmine inhibited bladder cancer cell proliferation, and the Western blotting result showed that harmine could induce cell apoptosis in RT4 cells, which means that the expression level of cleaved PARP and caspase were all decreased (Figure 4A). Meanwhile, large number of events in angiogenesis such as endothelial cell migration, proliferation, and formation of tubes are mainly mediated by VEGFR2 activated by VEGF and its downstream signaling molecules [24,25]. To explore the molecular mechanism through which harmine exerted anti-angiogenic functions, we detected some key signaling molecules involved in VEGFR2-mediated signaling pathway. As shown in Figure 4B, harmine strongly inhibited VEGF-induced VEGFR2 phosphorylation at Tyr1175 site in a dose-dependent manner when endothelial cells were exposed to VEGF. Moreover, harmine suppressed the expression of p-AKT and p-ERK1/2, these proteins are the downstream signaling molecules of VEGFR2 and play a critical role in proliferation, migration, and differentiation of endothelial cells, indicating that harmine inhibited angiogenesis through VEGFR2-mediated pathway.

## Discussion

In the present study, a natural product named harmine was determined as a potent angiogenesis inhibitor in the curative prospect of bladder cancer treatment. Harmine inhibited VEGF-mediated tumor angiogenesis, cell proliferation, migration, invasion, and tube formation through multi-spectrum. And *in vivo* xenograft human bladder tumor mouse model strongly verified that harmine had the pharmacological activity in blocking tumor growth and tumor angiogenesis.

As the most common cancer of the uniray system, bladder cancer accounts for an incidence rate of 350000–380000 cases being reported per year [26]. Among all these cases, it can be mainly divided into two clinical phenotypes: the first type is the non-muscle-invasive bladder cancer and the second type is the muscle-invasive bladder cancer. Non-muscle-invasive cancers frequently recur at approximate rate of 50–70% [27]. Muscle-invasive cancers have a 5-year survival rate of <50% [28]. With a poor prognosis, there is an urgent need to develop new drugs and to look for new drug targets for bladder cancer treatment. Some literature has revealed that VEGF receptor is a promising class of cancer treatment drug target including bladder cancer [11]. VEGF plays the most important role in the process of tumor angiogenesis. It stimulates multiple downstream signals in the cell and these stimulation involve many tyrosine kinases and its target molecules. All these signal molecules may have a relationship with tumor angiogenesis, endothelial cell survival, cell proliferation, migration, and vascular permeability [5]. In clinic, about more than 20 kinds of drugs have been used for anti-angiogenics. These drugs include VEGF-neutralizing antibody, soluble receptors, receptor antagonists, and some tyrosine kinase inhibitors of which some were undergoing clinical (Phase I–III) trials and some already approved for cancer treatment [29–31].

Here, we identified that harmine has anti-angiogenetic efficacy when it shows the inhibition on bladder cancer growth. Harmine obviously impeded the kinase activity of VEGFR2, suggesting that harmine might be a potential VEGFR2 kinase antagonist. For the bladder cancer, harmine shows its therapeutic capacity partially due to its inhibition on VEGFR2. And further molecular mechanism of harmine against bladder cancer needs more research.

In a word, our study provides evidence that harmine has a great application prospect in bladder cancer treatment.

## Abbreviations

FBS: fetal bovine serum
HUVEC: human umbilical vein endothelial cell
p-VEGFR: phospho-VEGFR
VEGF: vascular endothelial growth factor
VEGFR: vascular endothelial growth factor receptor
